# Viewing Time Measures of Sexual Interest and Sexual Offending Propensity: An Online Survey of Fathers

**DOI:** 10.1007/s10508-022-02324-5

**Published:** 2022-10-04

**Authors:** Patrizia Pezzoli, Kelly Babchishin, Lesleigh Pullman, Michael C. Seto

**Affiliations:** 1 Division of Psychology and Language Sciences, Faculty of Brain Sciences, University College London, 26 Bedford Way, London, WC1H 0AP UK; 2 University of Ottawa’s Institute of Mental Health Research at The Royal, Ottawa, ON Canada; 3 Department of Psychology, Carleton University, Ottawa, ON Canada

**Keywords:** Sexual offending, Viewing times, Sexual interest, Incest, Pedophilia, DSM-5-TR

## Abstract

Relative viewing times (VTs)—time required to view and evaluate sexually salient images—discriminate individuals with a sexual interest in children, as indirectly indexed by their history of sexual offending against children, from those without such history. In an online sample of 652 fathers, we measured VTs and sexual attraction ratings to child and adult images. We assessed participants’ sexual offending history and propensity (self-reported likelihood to have a sexual contact with a child, a non-consensual sexual contact with an adult, and propensity toward father–daughter incest). In contrast with VT studies involving clinical or forensic samples, VTs and attraction ratings failed to discriminate participants with a sexual offending history. VTs successfully distinguished participants with a propensity to sexually offend against children but failed to identify those with a propensity toward incest. Conversely, attraction ratings distinguished participants with a propensity toward incest but failed to identify those with a propensity to sexually offend against children. Correlations between VTs and attraction ratings were small. Results illustrate, for the first time, the distribution of VT measures in community fathers, support the feasibility of online administration of VT tasks to detect propensity to sexually offend against children, and indicate that sexual interest in children and incest propensity are distinct.

**Supplementary Information:**

The online version contains supplementary material available at 10.1007/s10508-022-02324-5.

## Introduction

Sexual interest in children is a major risk factor for the onset and maintenance of sexual offending against children by men (Hanson & Morton-Bourgon, 2005; Seto, 2019; Whitaker et al., 2008). Since not all men who share this interest report engaging in sexual behavior involving children (Bailey et al., 2016; Beier, 2021), sexual interest in children is not synonymous with sexual offending. Nevertheless, sexual interest in children is a valid and reliable predictor of sexual offending (Seto, 2018; Whitaker et al., 2008). As such, a large body of research has tried to identify ways to successfully measure male sexual interest in children (Carvalho et al., 2020). Historically, sexual interest has been measured using self-report questionnaires or penile plethysmography—a physiological method to directly measure genital sexual arousal (Seto, 2018)—but both methods present limitations. Most importantly, self-reports are vulnerable to socially desirable responding, especially when there are serious legal or other repercussions (Kalmus & Beech, 2005; Seto, 2018). Penile plethysmography is still subject to conscious manipulation and is also more expensive and intrusive (Wilson & Miner, 2016). As a result, researchers have developed indirect methods to assess sexual interests, including relative viewing time (VT) measures (Pedneault et al., 2021; Schmidt et al., 2017).

### Viewing Time Measures of Sexual Interest in Children

VT measures are based on the notion that preferred sexual stimuli are gazed at longer than non-preferred sexual stimuli (Schmidt et al., 2017). Proposed explanations of this phenomenon include: (1) interference with information processing, referred to as sexual content-induced delay (Geer & Bellard, 1996; Imhoff et al., 2020) or as hot cognitive processes (Imhoff et al., 2010) and (2) deliberative mate selection processes or internal reminiscence of preferred sexual stimuli (Wilson & Miner, 2016), also referred to as cold processes (Imhoff et al., 2010). Sexual content-induced delay (i.e., hot cognitive processes) describes the delay as being explained by the preferred sexual stimuli triggering distracting or resource-intensive cognitive processes. In contrast, deliberative mate selection processes (i.e., cold cognitive processes) describes the delay as being explained by the length of time it takes to appraise the sexual attractiveness of the stimuli (i.e., feature-checking), with longer delay for attractive stimuli, as there are more features (e.g., gender, physical appearance) to be assessed than unattractive stimuli. Using variants of VTs, Schmidt et al. (2021) found that VT effects could be explained by the cold cognitive process hypothesis that more information is required to process sexually preferred than non-preferred targets, resulting in longer VTs (see also Imhoff et al., 2012; Pohl et al., 2016).

Since their introduction, VT measures of sexual interest have been predominantly used to investigate sexual orientation for gender (e.g., Israel & Strassberg, 2007; Rönspies et al., 2015). However, VTs have also been used to assess sexual preferences in terms of age and are one of the most common measures of sexual interest in children used in forensic setting (McGrath et al., 2010). This practice is based on the assumption that a history of sexual offending against children can be a proxy—albeit imperfect—for sexual interest in children, which is what VTs ultimately aim to detect. VTs have been shown to moderately discriminate men with compared to without a history of sexually offending against children, and to have small-to-moderate convergent validity with self-reports, penile plethysmography, and other indirect measures of sexual interest in children (Pedneault et al., 2021; Schmidt et al., 2017). Further supporting the construct validity of VT measures, one study found that VTs for child stimuli significantly predicted sexual recidivism among men who had sexually offended (Gray et al., 2015).

### Sexual Interest in Children and Incest Offending

VT measures have been designed to address sexual interest and, therefore, may relate to sexual behavior—such as sexual offending—when that behavior is motivated by such interests, rather than other pertinent factors. For example, while sexual interest in children is an important motivation for sexual offending against children overall, it does not help explain most incest offending, because most incest perpetrators do not show greater sexual interest in children than adults (Seto, 2019). Incest perpetrators score lower on measures of sexual interest in children relative to extrafamilial perpetrators with child victims (Seto et al., 2015), including indirect measures such as VT tasks (Schmidt et al., 2014). This is counterintuitive because greater sexual interest in children has been hypothesized as a cause of incest offending, to overcome the effects of biological incest aversion and social norms against incest (Antfolk et al., 2012; Pullman et al., 2017; Seto, 2008, 2018).

It is possible that the difference in the importance of sexual interest in children between incest and extrafamilial perpetrators reflects a bias in extant literature due to the composition of clinical or forensic samples. For example, we might expect the results described in Seto et al. (2015) if all incest perpetrators—whether scoring high or low on sexual interest in children—are referred for evaluations to determine the risk they might pose to other children in the family, but if only extrafamilial perpetrators with particularly young victims or multiple child victims—indicators of high sexual interest in children—are referred. Moreover, it is possible that risk factors other than sexual interest in children, such as uncertainty about the kinship of one’s child (Seto et al., 2015), predispose some individuals to incest offending. Population-based studies investigating whether sexual interest in children, as measured by relative VTs, predicts propensity to sexual offend against children but not incest offending, can therefore help to solve this puzzle.

### The Present Study

Although VTs appear to be a promising tool to measure sexual interest in children, normative data are lacking (Gress, 2005; Schmidt et al., 2017). First, no prior study has demonstrated that sexual interest for an age category, as measured by VTs, relates to the propensity to sexually offend against individuals in that specific age category. Second, no study has investigated this relationship in a community sample of fathers. The resulting lack of normative data on the link between VTs and sexual interest leaves it unclear how unusual a particular VT pattern might be, therefore making it difficult to establish a critical score that can identify those who are likely to be attracted to children, based on their VT patterns (Seto, 2017a, b). For instance, one laboratory study found that men attracted to sexually mature adults *and* men attracted to children gazed longer at child compared to adult sexual stimuli (Pezzoli et al., 2020). Conceivably, child sexual stimuli can be highly salient in a non-sexual way (e.g., distressing or disturbing) and, as a result, capture the attention of both sexual interest groups alike. This may be particularly the case for fathers, whose brain activity in response to child and sexual stimuli differs from that of non-fathers (Mascaro et al., 2014). As a result, research needs to clarify how VTs for child and adult stimuli distribute in the general population and, especially, in fathers.

We examined, for the first time in an online community sample of fathers, the relationship between VTs for sexual stimuli and a history of or propensity toward sexual offending against children or adults, including incest. This empirical test was needed as VT patterns vary between individuals with a history of sexual offending versus individuals without a history of sexual offending and predict future sexual offending (Schmidt et al., 2017), but their ability to predict specific sexual offending behaviors, including incest, has never been tested. We focused on fathers with daughters as father–daughter incest is more common than father–son incest and offenses committed against boys are likely to involve sexual interest in children (Seto, 2018).

We administered the VT task online, as part of a larger survey, to explore its feasibility in this modality and increase potential recruitment. In the full sample of fathers—differentiated by genetic relatedness with their children—we explored the distribution of VTs and rated sexual attraction to child and adult visual sexual stimuli. We also examined the distribution of sexual offending histories and indicators of propensity to sexually offend against children, adults, and to engage in incest. Then, using receiver operating characteristic (ROC) curve analyses, we investigated whether VTs discriminated participants based on their sexual offending history and self-reported propensity to sexually offend. Based on data from clinical and forensic samples (Pedneault et al., 2021; Schmidt et al., 2017; Seto et al., 2015), we expected that VTs would show a moderate ability to discriminate participants based on their sexual offending history (Hypothesis 1) and propensity (Hypothesis 2), with longer VTs for the preferred age category in those with a history of or propensity toward sexual offending against children, compared to their counterparts, but no discrimination for incest propensity specifically (Hypothesis 3).

## Method

### Participants

The current study is a new analysis of data from a larger project aimed to elucidate the factors underlying incest propensity and behaviors using an online survey of fathers of daughters from the community (Pullman, 2018). No previous study examined VT data from the same sample, and the previous publication involving this sample used other measures to address specific hypotheses on sexual interests in community fathers. Participants were recruited using online advertisements linking to a survey implemented on the Qualtrics platform. Ethics approval was received from the University of Ottawa (protocol number H07-17-39) and the Royal Ottawa Health Care Group’s Research Ethics Board (protocol number 2015016). Data collection was conducted in two phases, one in November 2017 and one in February and March 2018, the latter targeting sociolegal fathers only, who were only 7% of the sample in the first wave. To take part in the study, participants had to be male, 18 years old or older, proficient in English, and a biological, step, or adoptive father to at least one daughter. Eligibility was established based on responses at the beginning of the survey. The survey examined fatherhood (e.g., time spent living with children), childhood history, and sexual interests and behaviors.

In total, 835 participants completed the survey. After validity screening, which involved excluding participants who rushed through the survey and who did not provide consistent responses to validity questions, we retained data from 652 participants for statistical analyses (78% of the full sample; please see Supplemental Material 1 for further details). The final sample (*N* = 652) comprised 224 fathers with biological children exclusively, 348 fathers with both biological and sociolegal children (279 step, 35 adoptive, 34 both), and 80 fathers with sociolegal children only (46 step, 28 adoptive, 6 both). Participants were predominantly middle-aged (age *M* = 42.7, SD = 11.6), college-educated (71% attended college or university), and with a medium-to-high income on average (75th percentile earned $80,000 or more in the previous year, before taxes). Of the 652 fathers, 74% resided in the USA and 26% in Canada. Demographic characteristics are reported in Table 1.

**Table 1:** Demographic characteristics of the sample

	*n* (%)			
	Sample size	Biological only (*n* = 224)	Biological and sociolegal (*n* = 348)	Sociolegal only (*n* = 80)
Education				
Up to completed high school	192 (29)	114 (51)	225 (65)	46 (58)
Some college to completed graduate school	460 (71)	110 (49)	123 (35)	34 (43)
Income				
Up to $50,000	341 (53)	110 (50)	191 (56)	40 (51)
$51,000 or more	302 (47)	110 (50)	153 (44)	39 (49)
Country of residence				
Canada	167 (26)	63 (28)	84 (24)	20 (25)
USA	485 (74)	161 (72)	264 (76)	60 (75)
Current marital status				
Not married	229 (35)	93 (42)	114 (33)	22 (28)
Married	420 (65)	130 (58)	232 (67)	58 (73)
Child sex				
Daughters only	228 (35)	99 (44)	80 (23)	49 (61)
Daughters and sons	424 (65)	125 (56)	268 (77)	31 (39)

Income is reported in US$. Nine participants did not report their income (4 fathers with exclusively biological children, 4 with biological and sociolegal children, 1 with exclusively sociolegal children) and 3 did not report their marital status (1 father with exclusively biological children, 2 with biological and sociolegal children), and percentages are adjusted accordingly

*Biological only* fathers with biological children exclusively, *Biological and sociolegal* fathers with both biological and sociolegal (step or adoptive) children, *Sociolegal only* fathers with sociolegal children exclusively

### Measures

#### Viewing Time Task: Attraction to and Viewing Times for Sexual Images

**Fig. 1:**
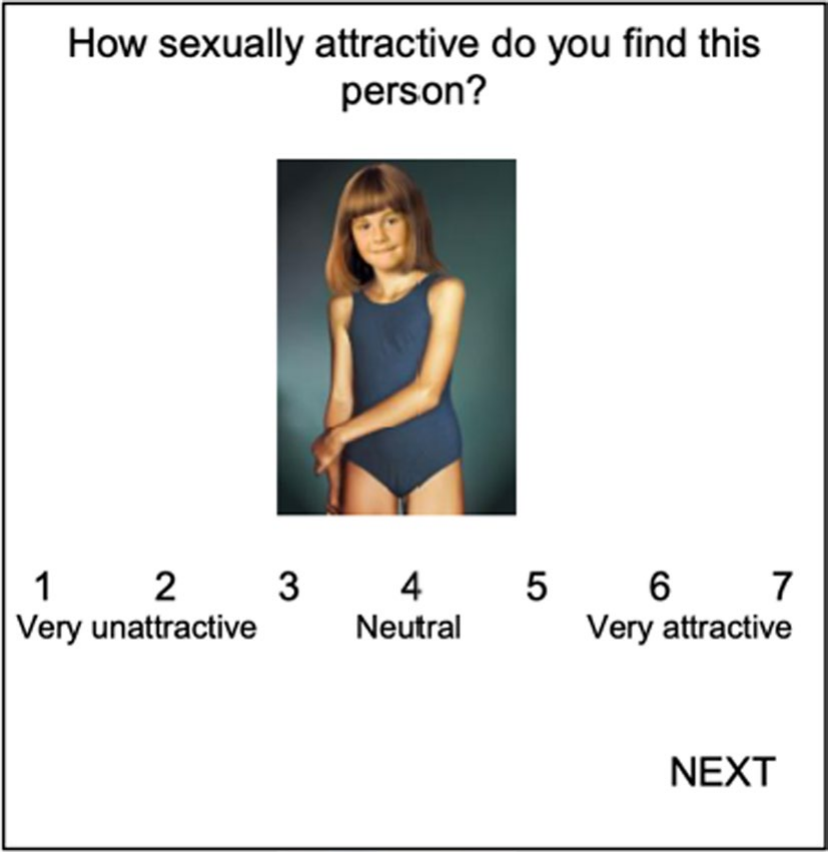
Example of a viewing time trial. Participants were instructed to view and evaluate the sexual attractiveness of visual sexual stimuli on a scale from very unattractive (1) to very attractive (7). During such trials, we unobtrusively measured their viewing times, namely the time to select an attractiveness rating before moving on to the next trial

We asked participants to view and rate on a 7-point scale the sexual attractiveness of 40 images depicting individuals wearing bathing suits across the 5 Tanner stages of sexual maturity (Tanner, 1990), namely, 4 stimuli per Tanner stage per gender. Images from the “Not Real People” picture set are real photographs that were digitally merged to create new images of people (NRP; Pacific Psychological Assessment Cooperation, 2004). While participants rated the attractiveness of the images, we unobtrusively measured their VTs (see Fig. 1 for a sample trial) using the built-in reaction time option in Qualtrics. In the current sample, Cronbach’s *α* for attraction ratings ranged from .95 to .98 for male images (Mdn = .97) and .77 to .96 for female images (Mdn = .93). Cronbach’s *α* for VTs (logged transformed) ranged from .74 to .78 for male images (Mdn = .75) and .71 to .78 for female images (Mdn = .74). Cronbach’s *α* for the VTs difference scores was .79 for the female pictures and .82 for the male pictures (see Supplemental Table 1).

Based on attraction ratings and on the time that participants took to provide them, we created absolute and relative attraction and VT scores. We computed absolute attraction scores by averaging ratings for 24 child (Tanner stages 1 through 3) and 16 adult (Tanner stages 4 and 5) female and male stimuli separately and then selecting the highest-rated age category across stimulus gender. Similarly, we created absolute VT scores by computing the average VTs for 24 child (Tanner stages 1 through 3) and 16 adult (Tanner stages 4 and 5) female and male stimuli separately and then selecting the longest average score across stimulus gender. We used absolute attraction and VT scores for descriptive analyses. We grouped images from Tanner stages 1 through 3 because sexual attraction to infants or toddlers (Stage 1) is very rare (Seto, 2016) and children in Stages 2 or 3 would mostly fall below legal ages of consent. Considering Tanner stages 1, 2, and 3 separately would decrease our statistical power to detect meaningful effects. For discrimination analyses, we computed relative attraction scores by subtracting the score for the highest-rated child category from the score for the highest-rated adult category. Similarly, we computed relative VT scores by subtracting the longest VTs for a child category from the longest VTs for an adult category. Positive relative scores thus indicate that participants rated adult stimuli as more attractive than child stimuli and gazed longer at adult relative to child stimuli, whereas negative scores indicate that participants rated as more attractive and gazed longer at child relative to adult stimuli.

#### Sexual Offending History

We asked participants whether they had ever been arrested, charged, or convicted of forcing someone to engage in sexual activity against his or her will (including someone who could not legally consent), of watching child pornography, as well as any non-contact sexual offenses (e.g., indecent exposure). We did not inquire about undetected criminal behavior to prevent concerns about self-incrimination or mandatory reporting obligations, given that IP addresses were automatically recorded by the Qualtrics server we used for this survey (though not shared with us, the researchers).

#### Sexual Offending Propensity

We obtained three indicators of sexual offending propensity. For the first two indicators, we asked participants to rate how likely they would be to engage in sexual contact with a child and how likely they would be to rape an adult, if assured of not being caught and punished, both on a scale from 1 (“not at all likely”) to 5 (“very likely”). For the third indicator, we asked participants to read four vignettes describing third-party sexual activity (genital fondling and intercourse) between a father and his daughter, adapted from Albrecht et al. (2014). Based on the premise of egocentric empathy, in which an individual experiences someone else’s behavior as if it were their own (Fessler & Navarrete, 2004), we asked participants to respond to three questions that would indicate a propensity for the behavior described. Specifically, questions addressed: (1) how sexually arousing they considered the story on a scale from 1 (“not at all arousing”) to 10 (“extremely arousing”); (2) how likely they thought that the man in the story would encourage continued sexual contact; and (3) how likely they would encourage continued sexual contact in a similar situation, on a scale from 1 (“not at all likely”) to 10 (“extremely likely”). For descriptive analyses, we computed three average scores, reflecting the average response to each question, across the four vignettes. For discrimination analyses, we obtained a single father–daughter incest propensity score by summing responses to these 12 items (3 items for each of the 4 vignettes, Cronbach’s *α* = .79), as in prior work on this data (Pullman, 2018).

### Statistical Analyses

#### Data Preparation

We prepared data for analyses using the R packages dvmisc (Van Domelen, 2019) and dplyr (Wickham et al., 2018). Data preparation included creating subsets of the sample by genetic relatedness with their children, logarithmically transforming (natural log) VT scores, all of which were non-normally distributed, normalizing absolute and relative attraction and VT scores, and outlier analysis. Removing outliers, defined as values 1.5 times the interquartile range less than the first quartile and greater than the third quartile (Field, 2013), did not affect results. Considering this, as well as because we used nonparametric tests and bootstrapping, which give outliers proportionally little weight (Gress et al., 2018), we report here results using the full data, outliers included. For discrimination analyses, given ceiling effects, we obtained a single score discriminating participants with vs. without sexual offending histories, and we dichotomized the three sexual offending propensity scores using a median split procedure (“not at all likely/arousing” vs. any other responses). This approach is considered acceptable with skewed distributions when analyzing group differences (Iacobucci et al., 2015).

#### Descriptive Analyses

We inspected the distribution of the variables of interest in the full sample and by genetic relatedness with their children. We inspected frequencies using the R package psych (Revelle, 2019), we computed zero-order Pearson correlations and compared subsamples in base R, using the Welch two sample *t* test for normally distributed variables (i.e., incest propensity, absolute ratings, and absolute VTs) and the Mann–Whitney–Wilcoxon test for non-normally distributed variables (i.e., offense histories, self-reported likelihood to have sexual contact with a child and to rape an adult). Distributions were plotted using ggplot2 (Wickham, 2016).

#### Discrimination Analyses

In order to inspect whether VTs discriminated fathers with any (officially detected) sexual offending history and sexual offending propensity from fathers without any sexual offending history and propensity, we computed the areas under the curve (AUCs) of the ROC and compared the AUC of paired ROC curves (bootstrap method) using the R package pROC (Robin et al., 2011). Relative—rather than absolute—VT scores were used as relative scores show greater validity (Schmidt et al., 2017). We opted for the ROC approach over logistic regression due to its robustness to low base rates and deviations from the ideal half split (Babchishin & Helmus, 2015). In the risk assessment literature (e.g., Rice & Harris, 2005), it has been suggested that AUC values between .50 and .55 correspond to a small effect size (Cohen’s *d* = .20), values between .56 and 65 correspond to a medium effect size (*d* = .50), and values greater than .65 to a large effect size (*d* = .80). In the VT literature specifically, VT measures have been found to discriminate men who sexually offended against children from various offending and non-offending groups at *d*_fixed-effect meta-analysis_ = .52 to .84 (Mdn *d* = .56; Schmidt et al., 2017), which translates to AUCs of .64 to .72 (Mdn AUC = .65) using transformation tables in Salgado (2018).

## Results

**Fig. 2:**
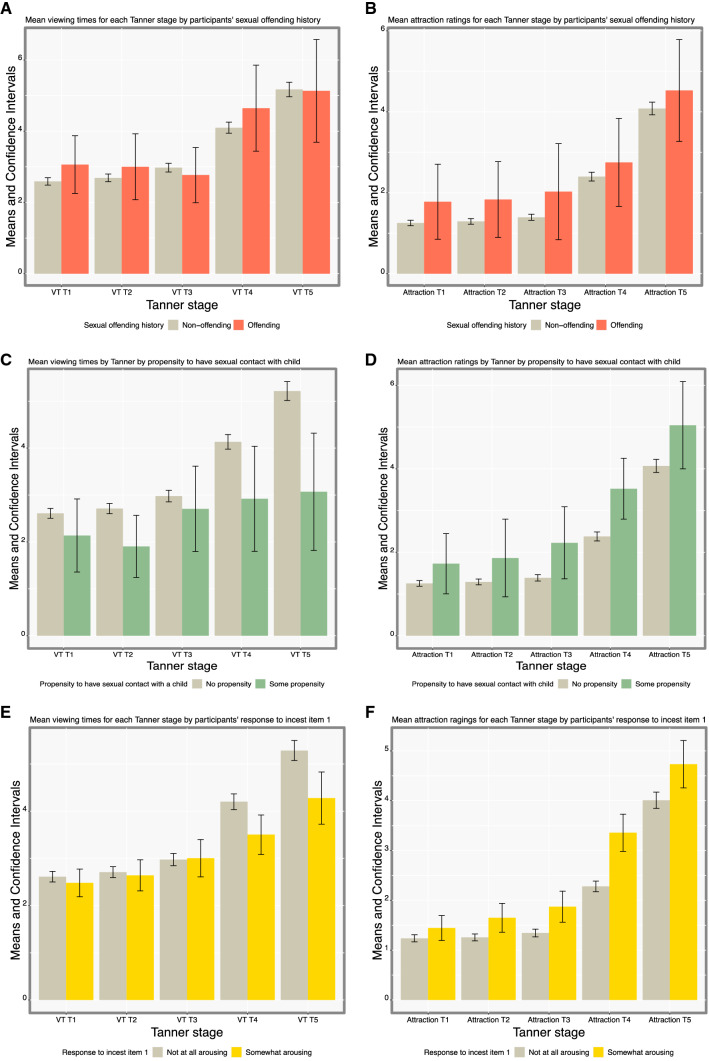

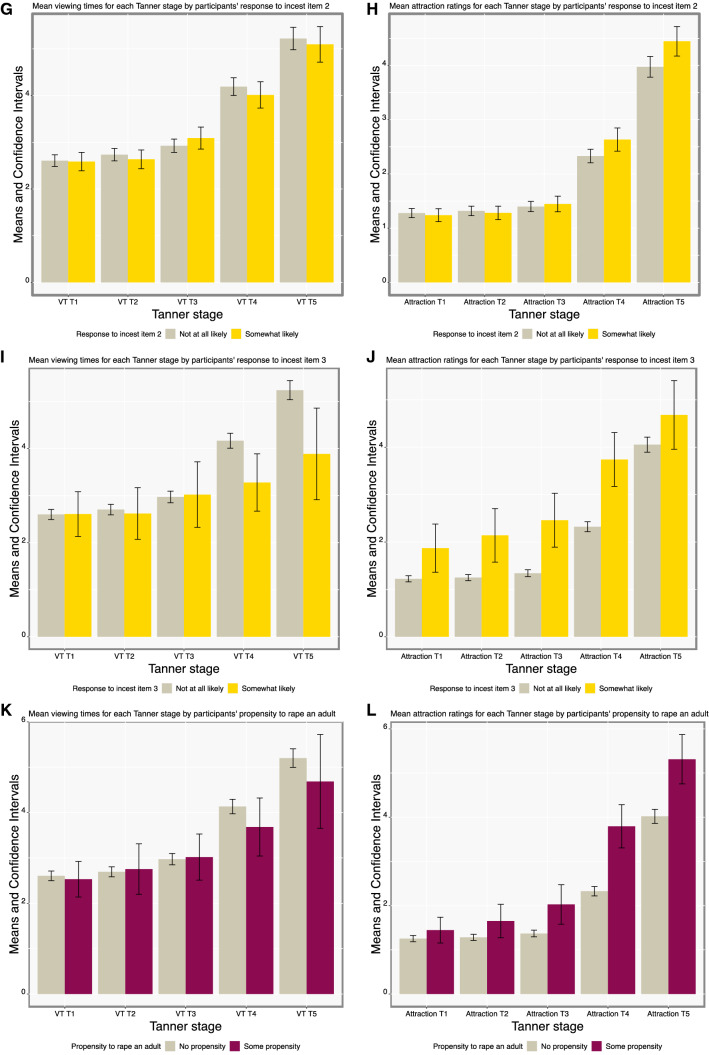
Average VTs (**A**) and average attraction ratings (**B**) for each Tanner stage by participants’ sexual offending history, average VTs (**C**) and attraction ratings (**D**) by Tanner stage by propensity to have sexual contact with a child, average VTs (**E**) and attraction ratings (**F**) by Tanner stage by response to incest item 1 (how sexually arousing they considered the story on a scale from “not at all arousing” to 10 “extremely arousing”), average VTs (**G**) and attraction ratings (**H**) by Tanner stage by response to incest item 2 (how likely they thought that the man in the story would encourage continued sexual contact on a scale from “not at all likely” to 10 “extremely likely”), average VTs (**I**) and attraction ratings (**J**) by Tanner stage by response to incest item 3 (how likely they would encourage continued sexual contact on a scale from “not at all likely” to 10 “extremely likely”), average VTs (**K**) and attraction ratings (**L**) by Tanner stage by propensity to rape an adult. Raw scores are reported here for ease of interpretation. Non-overlapping 95% CIs statistically significant at *p* < .01

Figure 2 illustrates the mean raw VTs and attraction ratings for each Tanner stage across stimulus gender, by participants’ sexual offending history and propensities.

### Sexual Offending History and Propensity

Less than 2% of fathers (*n* = 9, all fathers to both biological and sociolegal children) reported a history of sexual offending, whether for a contact (*n* = 7) or non-contact sexual offense (*n* = 2). No participant reported being arrested, charged, or convicted for child pornography. No significant difference was found between biological and sociolegal fathers on any of the indicators of sexual offending propensity (*ps* > .05). However, effect sizes were small-to-moderate, indicating that statistical significance could be achieved in larger samples (see Supplemental Table 2 for test statistics results and measures of effect size). Virtually all participants reported being “not at all likely” to engage in sexual contact with a child (98%; *n* = 639) and 2% reported being either “unlikely” (*n* = 7), “neutral” (*n* = 3), “likely” (*n* = 1), or “very likely” to (*n* = 2). Most respondents reported finding incest vignettes “not at all arousing” (88%; *n* = 564)—considered the man in the story “not at all likely” to encourage continued sexual contact with his daughter (69%; *n*=437), and reported that they would be “not at all likely” to encourage continued sexual contact with their own daughters in a similar situation (95%; *n* = 609. Most respondents reported being “not at all likely” to rape an adult (94%; *n* = 613) whereas 6% of them reported being “unlikely” (*n* = 24), “neutral” (*n* = 6), “likely” (*n* = 6), or “very likely” to (*n* = 2).

**Table 2:** Viewing times (VTs) and attraction scores = average VTs (in seconds) and attraction ratings (on a 1 to 7 scale) for sexual stimuli in the corresponding age category. Relative VT and attraction scores = difference scores computed as adult–child, so that a positive relative scores indicates that participants gazed longer at adult relative to child stimuli and rated them as more attractive, and vice versa for negative scores. Incest propensity (arousal) = average ratings (on a 1 to 10 scale) of how sexually arousing participants considered incest vignettes; Incest propensity (third person) = average ratings (on a 1 to 10 scale) of how likely they thought that men in the vignettes would encourage continued sexual contact with their daughters; Incest propensity (first person) = average ratings (on a 1 to 10 scale) of how likely participants themselves would encourage continued sexual contact with their own daughter in a similar situation

	*M* (SD)														
	Full sample	Genetic relatedness		Sexual offending history		Likelihood to have sexual contact with a child		Incest propensity (arousal)		Incest propensity (third person)		Incest propensity (first person)		Likelihood of raping an adult	
		Any biological (*n* = 572)	Sociolegal only (*n* = 80)	No (*n* = 643)	Yes (*n* = 9)	Not at all likely (*n* = 639)	Somewhat likely (*n* = 13)	Not at all arousing (*n* = 564)	Somewhat arousing (*n* = 78)	Not at all likely (*n* = 437)	Somewhat likely (*n* = 192)	Not at all likely (*n* = 609)	Somewhat likely (*n* = 34)	Not at all likely (*n* = 613)	Somewhat likely (*n* = 38)
VTs for child stimuli	4.77 (9.19)	4.90 (9.73)	3.81 (3.34)	4.79 (9.25)	3.02 (0.94)	4.77 (9.25)	4.45 (6.09)	4.62 (9.15)	5.88 (9.99)	4.8 (10.15)	4.78 (7.21)	4.76 (9.44)	5.00 (4.80)	4.79 (9.42)	4.53 (4.19)
VTs for adult stimuli	6.72 (10.66)	6.73 (11.06)	6.71 (7.24)	6.75 (10.73)	5.08 (1.63)	6.79 (10.76)	3.47 (1.74)	6.83 (11.28)	6.04 (5.25)	6.71 (12.10)	6.9 (7.15)	6.73 (10.89)	6.68 (7.09)	6.83 (10.97)	5.11 (2.81)
Attraction to child stimuli	1.38 (0.89)	1.37 (0.89)	1.41 (0.90)	1.37 (0.88)	1.89 (1.30)	1.36 (0.87)	2.22 (1.35)	1.30 (0.80)	1.93 (1.26)	1.36 (0.88)	1.41 (0.89)	1.31 (0.79)	2.53 (1.51)	1.35 (0.86)	1.89 (1.14)
Attraction to adult stimuli	3.32 (1.41)	3.33 (1.40)	3.30 (1.50)	3.32 (1.42)	3.85 (1.16)	3.30 (1.40)	4.55 (1.34)	3.20 (1.35)	4.28 (1.44)	3.25 (1.39)	3.58 (1.41)	3.26 (1.37)	4.44 (1.50)	3.24 (1.38)	4.7 (1.28)
Relative VT score	1.96 (9.72)	1.82 (9.97)	2.90 (7.63)	1.95 (9.78)	2.06 (1.50)	2.02 (9.78)*	− 0.98 (5.63)*	2.21 (9.72*)	0.16 (10.18)*	1.90 (10.12)	2.13 (9.35)	1.97 (9.93)	1.68 (6.48)	2.04 (9.97)	0.58 (3.74)
Relative attraction score	1.94 (1.30)	1.95 (1.30)	1.89 (1.27)	1.94 (1.30)	1.96 (1.07)	1.93 (1.29)	2.32 (1.76)	1.89 (1.28)**	2.35 (1.39)**	1.88 (1.31)*	2.16 (1.23)*	1.94 (1.29)	1.91 (1.46)	1.88 (1.28)**	2.81 (1.24)**

We report untransformed unstandardized VT and attraction scores here for ease of interpretation, but log-transformed standardized difference scores were used for the analyses. *Comparison significant at *p* < .05. **Comparison significant at *p* < .01. Test statistics and effect sizes are reported in Table 3 and Supplemental Table 1

### Viewing Times and Attraction Ratings

Raw VTs and attraction ratings are reported in Supplemental Table 3. Means and SDs for VT and attraction scores in the full sample, in subsamples by genetic relatedness with their children, as well as in subsamples by sexual offending history and propensities are reported in Table 2. The majority of participants gazed longer at adult relative to child stimuli (86%; *n* = 559) and rated them as more attractive (99% of respondents; *n* = 617). Most gazed longer at female compared to male stimuli (83%; *n* = 543) and rated them as more attractive (86%; *n* = 536). There was a small and significant correlation between absolute VTs and attraction ratings for child stimuli (*r* = .17 [.10, .25], *p* < .001) but not for absolute VTs and attraction ratings for adult stimuli (*r* = .08 [− .01, .17], *p* = .070). There was also a small significant correlation between the relative VT and the relative attraction score (*r* = .12 [.02, .21], *p* = .010).

**Table 3:** Results of discrimination analyses

IV	DV	ROC curve analyses						Group comparisons				
		AUC	CI_L_	CI_U_	*p*_AUC_	*D*	*p*_*D*_	*W*	*d*	*d*_L_	*d*_U_	*p*_*W*_
Genetic relatedness with children	VT	*.52*	*.44*	*.60*	.66	.88	.38	15,901	− .14	− .37	.10	.64
	Attraction	.49	.42	.56	.77			20,934	.05	− .20	.29	.77
Sexual offending history	VT	.51	.34	.69	.90	− .43	.67	2254	− .01	− .67	.65	. 91
	Attraction	.50	.36	.66	.98			2747	− .02	− .67	.64	.98
Likelihood to have sexual contact with a child	VT	.74	.53	.89	.005**	.75	.46	3314	.31	− .24	.86	.01*
	Attraction	.58	.38	.78	.41			3294.5	− .30	− .85	.25	.30
Incest propensity	VT	.51	.46	.56	.42	− 1.46	.14	34,591	− .04	− .22	.14	.41
	Attraction	.58	.53	.63	6.86E−04**			34,536	− .29	− .46	− .12	9.22E−04**
Likelihood to rape an adult	VT	.57	.46	.74	.20	− 1.35	.18	7373	.15	− .18	.48	.21
	Attraction	.71	.62	.80	4.58E−06**			6476	− .73	− 1.06	− .40	1.73E−05**

*IV* independent variable, *DV* dependent variable, *VT* relative VT score, *Attraction* relative attraction score, *AUC* area under the curve, *CI*_*L*_ confidence intervals, lower bound, *CI*_*U*_ confidence intervals, upper bound, *p*_*AUC*_* p* value associated with the AUC, *D* difference between the AUC of the two measures (VT scores vs. attraction scores) divided by the standard deviation of the bootstrap differences, indicating whether one of the two measures is a better discriminator, *p*_*D*_* p* value associated with *D*, *W* Mann–Whitney nonparametric test comparing the corresponding groups on the relative VTs and relative attraction scores, *d* Cohen’s *d* effect size for the group comparison on the relative VTs and relative attraction scores, *d*_*L*_ lower bound, *d*_*U*_ upper bound, *p*_*W*_* p* value associated with *W*

**Comparison significant at *p* < .01, *comparison significant at *p* < .05

### Discrimination Results

The ROC graphs illustrating the ability of VT and attraction scores to discriminate participants based on the genetic relatedness with their children, their sexual offending history, and propensity are depicted in Supplemental Fig. 1; numerical results are reported in Table 3. Biological and sociolegal fathers did not significantly differ on VTs or attraction scores (AUC = .52 [.44, .60], *p* = .657 and AUC = .49 [.42, .56], *p* = .770, respectively). VTs and attraction scores also did not significantly discriminate the 9 participants who reported a sexual offending history from participants without a sexual offending history (AUC = .51 [.34, .69], *p* = .900 and AUC = .50 [.36, .66], *p* = .980, respectively). VTs were highly discriminant for those “not at all likely” to have a sexual contact with a child relative to those who provided any other response (AUC = .74 [.53, .89], *p* = .005), whereas attraction scores were not (AUC = .58 [.38, .78], *p* = .413). As illustrated in Fig. 2C, participants “not at all likely” to have a sexual contact with a child displayed increasing VTs as Tanner stages increased, whereas this effect was not observed in those somewhat likely to have a sexual contact with a child, leading to a smaller relative VT score in this group. While VTs failed to discriminate participants with some compared to little-or-no incest propensity (AUC = .51 [.46, .56], *p* = .416), attraction scores discriminated these participants moderately (AUC = .58 [.53, .63], *p* < .001). This result was in the direction opposite to what we expected: Participants who reported more incest propensity rated child stimuli as less attractive. Lastly, VTs failed to discriminate participants somewhat likely vs. “not at all likely” to rape an adult (AUC = .57 [.46, .74], *p* = .198), while attraction ratings were highly discriminant (AUC = .71 [.62, .80], *p* < .001). Participants who reported some likelihood to rape an adult (vs. being “not at all likely”) rated adult stimuli as relatively more attractive than child stimuli.

## Discussion

### The Utility of Viewing Time Measures to Discriminate Participants by Sexual Offending History and Propensity

In contrast with our first hypothesis as well as with prior studies involving offending men, VTs and attraction ratings for sexually salient images did not identify participants who reported they had committed a detected sexual offense from those who reported no such history. Because ROC curve analyses are appropriate with low base rates (Babchishin & Helmus, 2015), this nonsignificant result is unlikely explained by limited statistical power. Rather, differences in how offending status was determined in our study might contribute to the discrepancy with prior literature. Specifically, while VTs have been shown to moderately discriminate sexual offenders against children from various offending and non-offending groups (*d* fixed-effect meta-analysis = .52–.84; Schmidt et al., 2017, which translates to AUCs of .64–.72; Salgado, 2018), we addressed the ability of VTs to discriminate fathers with a sexual offending history involving victims of any age (children and/or adults). Breaking down the sample by victim age is thus important in future studies to clarify the utility of VTs in detecting different offending histories and would require a much large sample size of fathers than the current study (*N* = 652).

Consistent with our second and third hypotheses, results supported the ability of VTs to detect participants’ propensity to sexually offend against children, but not to father–daughter incest. Conversely, attraction ratings discriminated participants with some incest propensity (who, notably, rated child stimuli as *less* attractive) but failed to distinguish those with a propensity to sexually offend against children. Notably, VTs discriminated those with a propensity to have a sexual contact with a child because, compared to those with no such propensity, they did not display an increase in VTs for adult stimuli, not because they gazed longer at child stimuli. This result might reflect relatively reduced sexual interest in adults in this group, a possibility that has received some empirical support (Knott et al., 2016; Walter et al., 2007). Alternative explanations for this result (e.g., greater measurement error in this group or stimulus category) appear less plausible, as any methodological artifacts would affect responses to all trials equally. Of note, we used the built-in timer option in Qualtrics to collect VTs. Previous studies suggested that the built-in timer can be impacted by variation in noise due to the type of computers or browsers used and, as such, future studies could include Javascript or QRTEngine solutions that might offer more reliable measurement at the millisecond level (Barnhoorn et al., 2015). The failure of VTs to discriminate participants based on their incest propensity, as well as the fact that participants with greater incest propensity rated child stimuli as less attractive, is consistent with the finding that incest propensity is not adequately explained by sexual interest in children (Seto, 2018). Evidence from forensic and clinical samples indicates that most incest perpetrators do not report greater sexual interest in children than in adults and show less sexual interest in children than extrafamilial perpetrators with child victims (Seto et al., 2015). Our results indicate that the relation between sexual interest in children and incest propensity can be poor also in community men.

Our results also indicated a small correlation between VTs and attraction ratings. One in a 100 fathers rated child stimuli as more attractive than adult stimuli, which is unsurprising given how rare sexual preference for children is in the general population (e.g., 0.1% in Dombert et al., 2016). In contrast, more than one in ten participants gazed longer at child relative to adult stimuli, and VTs were related to self-reported propensity to sexual offend against a child (whereas attraction ratings were not). These results indicate that VTs can be a valid assessment of sexual interest in children among fathers. The correlation between relative VTs and relative attraction ratings estimated in our study (*r* = .12) was smaller than that found in VT studies involving offending men, where convergent validity between these indicators was small-to-moderate (*r* = .38 in Schmidt et al., 2017). Both offending and community men might be reluctant to disclosing their sexual attraction to children, albeit for different reasons (e.g., fear of legal repercussions and social desirability, respectively) and this might explain the low concordance. Alternatively, and as argued in a previous study (Pezzoli et al., 2020), it is possible that sexually salient images of children might capture participants’ attention more than sexually salient images of adults due to their surprising or disturbing nature, therefore prolonging VTs. In other words, we cannot directly discern whether some fathers gazed longer at child images because of their sexual interest in children or, rather, because those images were particularly surprising or disturbing. Since this interpretation is in contrast with the empirically supported cold cognitive processes explanation of VT effects (e.g., Schmidt et al., 2021), future VT studies involving fathers could help clarifying whether this explanation holds in fathers specifically.

In addition, we found that attraction ratings—but not VTs—discriminated participants with some propensity to sexually offend against adults. This result might reflect a preference for sexually salient images of adults in community men who are also more prone to engaging in sexually coercive behavior involving adults, although this preference was not reflected in longer VTs. Future studies are needed to determine whether VT tasks can be used to detect interest in sexual offending against adults, as well as other sexual interests (see also, Lalumière et al., 2018), particularly paraphilias, which are also linked to various forms of sexual offending (Seto, 2017a, b). For instance, individuals who report a fetishistic interest may display relatively longer VTs for fetish objects than neutral or other sexual stimuli, compared to those who do not. Consistent with this possibility, one prior study found that VT measures related to sadomasochistic sexual interest (Larue et al., 2014).

### Limitations and Recommendations for Future Research

While our online survey allowed us to collect information from a larger sample than in most prior VT studies (*M*_*N*_ = 159; Schmidt et al., 2017), the base rate for offending history and propensity in our study was still very low. In light of this, future studies should aim to recruit even larger samples, or to intentionally recruit non-representative samples (e.g., oversampling fathers with sexual offenses against children, fathers who sexually victimized their biological or sociolegal children). We did not ask participants with a sexual offending history whether they had child or adult victims (or both), which hinders our comparisons with VT studies involving offending samples. We also did not ask participants whether they ever engaged in sexually coercive behaviors that remained undetected by law enforcement, potentially biasing our sample split by sexual offending history. Prior studies indicated that as many as 18.5% of community men engage in any sexually coercive behavior (Baur et al., 2016) and 3% engage in sexual behavior involving children specifically (Dombert et al., 2016). The possibility of undetected offending behaviors in our sample is supported by participants’ responses to items explicitly addressing their likelihood to sexually offend. Namely, 6% reported some likelihood to rape an adult, 2% reported some likelihood to sexually offend against children, and 5% reported some likelihood to engage in incestuous behavior with their own daughter in a given situation. Therefore, future population-based VT studies should measure detected as well as undetected sexually coercive behaviors through the use of software programs that do not collect IP addresses, and further stratify participants by victim age. While self-reports of sexual offending behavior may be ill-suited to reliably detect sexual interest in children in population samples, propensity measures seem more promising. Moreover, to replicate evidence that VT effects reflect cold cognitive processes rather than sexual arousal regulation problems (Schmidt et al., 2021) future studies should include indicators of sexual arousal regulation, such as frequency of sexual activity or sexual risk taking.

While our study involved the largest community sample of fathers in the VT literature, it could not rule out self-selection and, as a result, potential bias in terms of demographic characteristics and outcomes of interest. Our participants were more educated than the general population on average (i.e., 31.6% had at least a college diploma, vs. 22.4% of the general population of Canada; Statistics Canada, 2016). We did not collect information on participants’ race and ethnicity and, thus, we were unable to break down our results accordingly. The online advertisement method might have influenced the demographic composition of the sample, and other recruitment strategies (e.g., market research panels) could be used to enhance representativeness in future studies. Future VT studies could address this limitation by recruiting fathers from population registries (e.g., Scandinavian countries). However, even in such studies, self-selection would exist. As a result, cumulative knowledge (Imhoff, 2020) based on evidence from multiple methodologies (online surveys, registry-based surveys) is required to conclusively determine the validity of online VT measures of sexual interest in children. The empirical evidence presented in this study represents an essential building block in generating this cumulative knowledge.

Factors relating to our procedure might have also influenced the results. First, the vignettes used to address incest propensity did not specify the age of the daughter in the story. As a result, our participants could have assumed that the daughter was postpubescent, making our vignettes a measure of incest propensity even without a sexual interest in children. Creating vignettes depicting prepubescent and postpubescent daughters in future studies would be preferable to separate measures of “pure” incest propensity from measures of sexual interest in children. Second, since our participants viewed images online (vs. in a controlled laboratory setting), bandwidth, browser, processor performance issues, as well as distracting environments could have induced unwanted lags. In line with this possibility, our participants viewed sexually salient images for 5.7 s on average, which is longer than the average VTs observed in previous studies involving offending men (3–4 s; Gress et al., 2011). Nonetheless, since every participant presumably completed the survey on one device from one location, technological and environmental factors might have introduced random noise rather than a systematic bias (e.g., biological fathers having slower internet speeds than sociolegal fathers). Also, participants might have gazed longer at the images because they were oblivious of their VTs being recorded, whereas participants in a laboratory might be more concerned about being observed. Differences between forensic and community samples in terms of access to pornographic material could have also played a role. While longer VTs could be expected in forensic patients, who have no other occasions to view sexually salient material, our results indicate that they might, in fact, require less time than community men to evaluate the attractiveness of this material.

### Conclusions

In an online sample of community fathers, we found that VTs were associated with self-reported propensity to sexually offend against children, and attraction ratings with self-reported propensity to sexually offend against adults. VTs were not significantly associated with sexual offending history or propensity to commit incest. If replicated, evidence that VT measures predict propensity to sexually offend against children speaks in favor of their applied use (e.g., to evaluate efficacy of prevention programs). While replication studies applying VT paradigms to online platforms in large population samples are warranted, our findings also support the feasibility of online administration of VT tasks to detect propensity to sexually offend against children. This evidence thus encourages future research aimed to further validate the use of online VT measures of sexual interest. Our study also adds to the literature that sexual interest in children and incest propensity are distinct and emphasizes the importance of collecting community as well as clinical or forensic VT data.

## Supplementary Information

Below is the link to the electronic supplementary material.

Supplementary file1 (DOCX 235 kb)

## Data Availability

The anonymized dataset, annotated scripts for analyses, and output files are available at the project’s webpage (osf.io/de5bk/).
