# Supplementary material for: Viewing Time Measures of Sexual Interest and Sexual Offending Propensity: An Online Survey of Fathers
Source: Arch Sex Behav. 2022 Oct 4;51(8):4097–110. doi: 10.1007/s10508-022-02324-5 (PMC9663406; doi:10.1007/s10508-022-02324-5)
Supplement: Supplementary file 1 — Supplementary file1 (DOCX 235 kb) [file 10508_2022_2324_MOESM1_ESM.docx]

**SUPPLEMENTAL MATERIAL**

### Validity Screening

Validity screening consisted of several steps. First, we excluded participants who rushed through the survey, meaning those who completing the survey in less than 7 minutes, which was approximately 1/3 of the median length of 20 minutes determined with a pilot. Second, we excluded participants who did not provide consistent responses to three validity questions. These questions included “Does five plus five equal ten?”, with “True” and “False” as possible responses; “Select a number between 30 and 35” from a 0-50 dropdown list; and to select the name of a character in a story presented. Third, we asked participants to report their age, the country where they currently lived, and their years of education twice, and excluded participants with inconsistent responses (allowing a variation of ±1 in age). We found no significant difference in the variables of interest between participants who did and did not pass the validity screening (see Pullman, 2018 for further details). We also analyzed the rate of missing data (i.e., “I prefer not to respond” choices) which was negligible (range: 0.15% – 3.98%, *M* = 1.39, *SD* = 1.74) and considered acceptable for valid statistical inferences (Dong & Peng, 2013).

**Reference**

Dong, Y., & Peng, C.-Y. J. (2013). *Principled missing data methods for researchers*. SpringerPlus, 2(1). doi:10.1186/2193-1801-2-222

### Supplemental Table 1

*Internal consistency of attraction ratings and viewing time composite scores*

| Tanner stage | Cronbach's alpha | | | |
| --- | --- | --- | --- | --- |
|  | Attraction ratings | | Viewing times | |
|  | Male | Female | Male | Female |
| 1 | .98 | .96 | .74 | .78 |
| 2 | .97 | .96 | .74 | .71 |
| 3 | .97 | .93 | .78 | .74 |
| 4 | .95 | .77 | .75 | .76 |
| 5 | .96 | .92 | .75 | .76 |
| Difference score | - | - | .82 | .79 |

*Note*. Viewing times (VTs) were log transformed (natural log) prior to analyses. We also computed 6 VTs difference scores (3 for each sex). First, we used the max viewing time of the female adult category (Tanner 4, 5) and subtracted the max viewing time of the Tanner 1 female child category. Second, we used the max viewing time of the female adult category (Tanner 4, 5) and subtracted the max viewing time of the Tanner 2 female child category. Last, we used the max viewing time of the female adult category (Tanner 4, 5) and subtracted the max viewing time of the Tanner 3 female child category. We then did the same for the male pictures.

### Supplemental Table 2

*Comparisons between biological fathers (n = 572) and sociolegal fathers (n = 80) on items measuring sexual offending propensity*

|  | Test statistic | *p* | *d* | *d_L_* | *d_U_* |
| --- | --- | --- | --- | --- | --- |
| Likelihood to have a sexual contact with a child | *W* = 22418 | .227 | -.20 | -.43 | .04 |
| Incest propensity (arousal) | *t* = 0.38 (108.70) | .706 | .29 | .06 | .52 |
| Incest propensity (third-person) | *t* = -1.20 (104.80) | .233 | -.14 | -.38 | .09 |
| Incest propensity (first-person) | *t* = -0.28 (99.36) | .782 | -.03 | -.27 | .20 |
| Likelihood to rape an adult | *W* = 23086 | .701 | .11 | -.12 | .35 |

*Note.* Sample size, means, and standard deviations are reported in Table 2S. Biological fathers were the reference group for *d*, so that a positive *d* indicates that biological fathers scored higher on the item than sociolegal fathers, and *vice versa* for a negative *d*. Incest propensity (arousal) = average ratings of how sexually arousing participants considered incest vignettes; Incest propensity (third-person) = average ratings of how likely they thought that men in the vignettes would encourage continued sexual contact with their daughters; Incest propensity (first-person) = average ratings of how likely participants themselves would encourage continued sexual contact with their own daughter in a similar situation; VT = Viewing time; *W* = Mann-Whitney test (non-parametric); *t* = Welch two sample t-test (degrees of freedom in parenthesis); *d* = Cohen’s d effect size; *d_L_* = Confidence interval, lower bound; *d_U_* = Confidence interval, upper bound. Note that 95% CIs for *d* were computed by formula, whereas CIs for *t*-tests were computed by bootstrap, which explains why the reported CIs may not include 0 even with a nonsignificant *t* test.

### Supplemental Table 3

*Mean raw viewing times and attraction ratings by each Tanner Stage, across stimulus gender, in the full sample and by participants’ sexual offending history and propensity*

|  |  | M (SD) | | | | | | | | | | | | |
| --- | --- | --- | --- | --- | --- | --- | --- | --- | --- | --- | --- | --- | --- | --- |
|  | Tanner stage | Full sample (n = 520) | Sexual Offending History | | Likelihood to have sexual contact with a child | | Incest propensity,  (arousal) | | Incest propensity,  (third-person) | | Incest propensity,  (first-person) | | Likelihood of raping an adult | |
|  |  |  | No  (n = 511) | Yes  (n = 9) | Not at all likely  (n = 509) | Somewhat likely  (n = 11) | Not at all arousing  (n =454) | Somewhat arousing  (n =58) | Not at all likely  (n =351) | Somewhat likely  (n =150) | Not at all likely  (n =351) | Somewhat likely  (n =150) | Not at all likely  (n = 491) | Somewhat likely  (n = 28) |
| Viewing times | 1 | 2.6 (1.20) | 2.59 (1.20) | 3.06 (1.05) | 2.61 (1.20) | 2.14 (1.16) | 2.61 (1.22) | 2.48 (1.11) | 2.61 (1.20) | 2.59 (1.22) | 2.61 (1.20) | 2.59 (1.22) | 2.61 (1.21) | 2.53 (1.01) |
|  | 2 | 2.7 (1.25) | 2.69 (1.25) | 3 (1.20) | 2.71 (1.25) | 1.90 (0.99) | 2.71 (1.26) | 2.64 (1.25) | 2.74 (1.26) | 2.64 (1.24) | 2.74 (1.26) | 2.64 (1.24) | 2.69 (1.24) | 2.75 (1.44) |
|  | 3 | 2.97 (1.41) | 2.98 (1.41) | 2.77 (1.01) | 2.98 (1.41) | 2.71 (1.35) | 2.97 (1.40) | 3.00 (1.50) | 2.93 (1.36) | 3.09 (1.46) | 2.93 (1.36) | 3.09 (1.46) | 2.97 (1.41) | 3.02 (1.31) |
|  | 4 | 4.11 (1.80) | 4.10 (1.80) | 4.65 (1.57) | 4.13 (1.79) | 2.92 (1.67) | 4.20 (1.80) | 3.5 (1.59) | 4.19 (1.81) | 4.01 (1.74) | 4.19 (1.81) | 4.01 (1.74) | 4.13 (1.80) | 3.68 (1.65) |
|  | 5 | 5.17 (2.33) | 5.17 (2.34) | 5.13 (1.87) | 5.22 (2.32) | 3.07 (1.86) | 5.28 (2.30) | 4.28 (2.10) | 5.22 (2.28) | 5.10 (2.37) | 5.22 (2.28) | 5.10 (2.37) | 5.20 (2.31) | 4.69 (2.67) |
| Attraction ratings | 1 | 1.26 (0.78) | 1.25 (0.77) | 1.78 (1.21) | 1.25 (0.77) | 1.73 (1.08) | 1.24 (0.75) | 1.45 (0.93) | 1.28 (0.80) | 1.24 (0.73) | 1.28 (0.80) | 1.24 (0.73) | 1.25 (0.78) | 1.45 (0.75) |
|  | 2 | 1.3 (0.79) | 1.29 (0.78) | 1.83 (1.22) | 1.29 (0.77) | 1.86 (1.38) | 1.26 (0.74) | 1.65 (1.07) | 1.32 (0.82) | 1.28 (0.76) | 1.32 (0.82) | 1.28 (0.76) | 1.28 (0.78) | 1.65 (0.98) |
|  | 3 | 1.41 (0.88) | 1.39 (0.86) | 2.03 (1.54) | 1.39 (0.86) | 2.23 (1.29) | 1.35 (0.83) | 1.87 (1.15) | 1.40 (0.90) | 1.45 (0.88) | 1.4 (0.90) | 1.45 (0.88) | 1.37 (0.85) | 2.03 (1.15) |
|  | 4 | 2.4 (1.23) | 2.4 (1.23) | 2.75 (1.41) | 2.38 (1.22) | 3.52 (1.09) | 2.28 (1.15) | 3.36 (1.39) | 2.33 (1.18) | 2.63 (1.31) | 2.33 (1.18) | 2.63 (1.31) | 2.33 (1.18) | 3.79 (1.26) |
|  | 5 | 4.09 (1.78) | 4.08 (1.79) | 4.53 (1.64) | 4.07 (1.78) | 5.05 (1.56) | 4.01 (1.77) | 4.73 (1.79) | 3.97 (1.81) | 4.45 (1.66) | 3.97 (1.81) | 4.45 (1.66) | 4.02 (1.78) | 5.31 (1.44) |

*Note*. The table presents data from a subset of the 652 participants retained after removing outliers from raw viewing times.

### Supplemental Figure 1


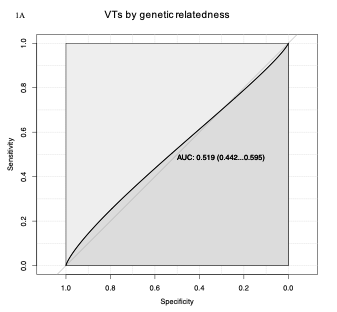

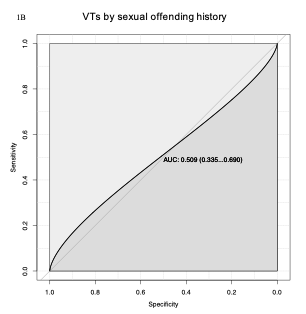


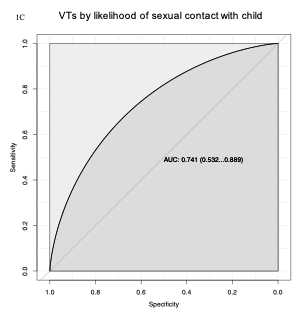

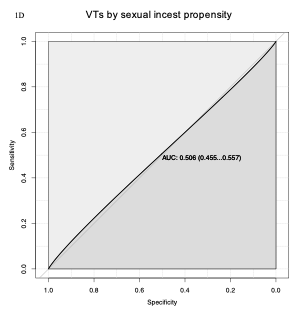


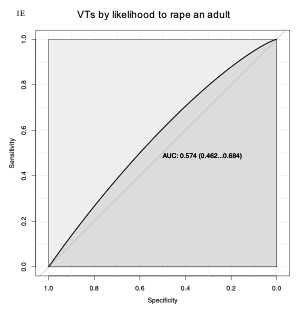

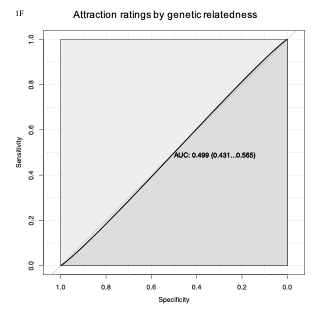


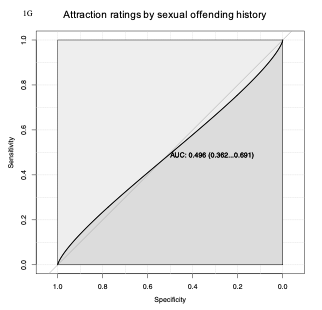

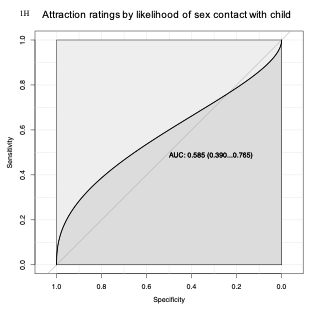


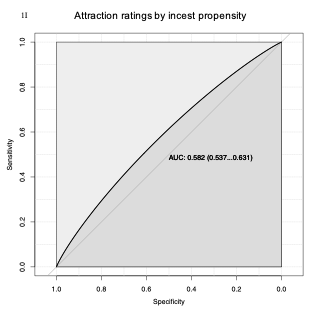

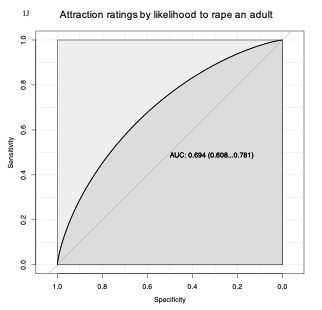


*Supplemental Figure 2S*. Receiver Operating Characteristic curves illustrating the ability of VTs (1A-1E) and attraction ratings (1F-1J) to discriminate groups by genetic relatedness (1A, 1F), sexual offending history (1B, 1G), likelihood to have a sexual contact with a child (1C, 1H); incest propensity (1D, 1I), and likelihood to rape an adult (1E, 1J). AUC values with 95% CIs not including .50 significant at *p* <.01. Small differences between the 95% CIs reported here vs. in the main text are due to different bootstrap runs.
